# Commencement

**Published:** 1877-04

**Authors:** 


### COMMENCEMENT.

The thirty-seventh annual commencement of the Baltimore College of Dental Surgery was held at the Academy of Music, Baltimore City, and was unusually interesting.

The exercises commenced with prayer, after which the Dean, Prof. F. J. S. Gorgas, D. D. S., M. D., announced the graduates, the authority by which the degree of "Doctor of Dental Surgery" was conferred, and the qualifications necessary.

The degree of Doctor of Dental Surgery was then conferred upon the following persons:

Pauline Boeck, Germany.
George Homer Bowman, Virginia.
Thomas Washington Crozier, Virginia.
Elias Drayton Earle, Florida.
Hammett Xavier Gale, Ohio.
Walter S. Harban, Maryland.
Sandy Stuart Harris, Virginia.

Robert Yates Henly, Jr., Virginia.
Thomas Huggins, England.
Frederick Koerner, Maryland.
Benjamin Lanier Lane, Georgia.
John E. La Motte, Maryland.
Luke Johnson Pearce, Maryland.
Charles Hector Reynolds, L. D. S., Canada.
James Matthews Roberts, Maryland.
James Edwin Shreeve, Maryland.
Henry Singruen, Germany.
Alonzo Stouch, Pennsylvania.
Marion William Williams, Tennessee.

The valedictory address was delivered by Dr. William H. Dwinelle, of New York City, and was received with marked attention by the large and brilliant audience present. This address contained pleasant and interesting references to the past thirty-seven year's history of the institution and the dental profession, and paid a glowing tribute to the memory of the founders of the Baltimore College of Dental Surgery.

After the close of the exercises at the Academy of Music, a large number of the alumni, members of the graduating class and invited guests, among whom were distinguished representatives of other professions, proceeded to the Masonic Temple, where the alumni supper was served, which proved a thoroughly enjoyable affair.

